# Supplementary material for: DNA Methylation Levels of the TBX5 Gene Promoter Are Associated with Congenital Septal Defects in Mexican Paediatric Patients
Source: Biology (Basel). 2022 Jan 8;11(1):96. doi: 10.3390/biology11010096 (PMC8773106; doi:10.3390/biology11010096)
Supplement: Supplementary file 1 [file biology-11-00096-s001.zip › biology-1512030-supplementary.pdf]

Supplementary Table S1. Exposure to environmental factors during pregnancy in mothers of patients with congenital septal defects.

Environmental factors	Congenital septal defects patients (n=35)	Controls (n=48)	*P*
**Drug addiction (%)**			0.566
Alcoholism	1 (2.9)	5 (10.4)	
Smoking	6 (17.1)	7 (14.6)	
Alcoholism /Smoking	0	1 (2.1)	
Unknown	0	0	
No exposure	28 (80)	35 (72.9)	
**Diseases during pregnancy (%)**			0.387
Preeclampsia	5 (14.3)	3 (6.3)	
Hypertension	3 (8.6)	3 (6.3)	
Gestational diabetes	3 (8.6)	1 (2.1)	
Diabetes mellitus type 1	0	1 (2.1)	
Diabetes mellitus type 2	2 (5.7)	1 (2.1)	
Epilepsy	0	0	
Rheumatoid arthritis	0	2 (4.2)	
Preeclampsia/DM T2	0	0	
H/DM T1	0	0	
DM T2/RA	0	1 (2.1)	
H/RA	0	0	
Unknown	0	1 (2.1)	
No exposure	22 (62.9)	35 (72.9)	
**Exposure to pollutants (%)**			0.993
Heavy metals	5 (14.3)	4 (8.3)	
Pesticides	3 (8.6)	8 (16.7)	
Solvents	6 (17.1)	2 (4.2)	
Herbicides	1 (2.9)	2 (4.2)	
Varnishes	0	3 (6.3)	
Unknown	0	0	
No exposure	20 (57.1)	29 (60.4)	
**Maternal infections (%)**			0.001
Urinary tract infection	10 (28.6)	21 (43.8)	
Vaginal candidiasis	11 (31.4)	9 (18.8)	
UTI/VC	8 (22.9)	0	
Unknown	0	0	
No exposure	6 (17.1)	18 (37.5)	
**Medication consumption (%)**			0.069
Nitrofurantoin	4 (11.4)	2 (4.2)	
Nitrofurantoin/Insulin	1 (2)	0	

Nitrofurantoin/Ampicillin	2 (5.7)	0	
Nitrofurantoin/ Nystatin	0	0	
Ampicillin	1 (2.9)	3 (6.3)	
NSAIDs	0	2 (4.2)	
NSAIDs/Nitrofurantoin	1 (2.9)	0	
Alfametildopa	3 (8.6)	5 (10.4)	
Alfametildopa/Ampicilin	2 (5.7)	0	
Alfametildopa/Hydralazine	0	1 (2.1)	
Alfametildopa/Nitrofurantoin	2 (5.7)	0	
Alfametildopa/Dexamethasone	2 (5.7)	0	
Nystatin	3 (8.6)	6 (12.5)	
Unknown	0	0	
No exposure	15 (42.9)	29 (60.4)	
**Consumption of vitamins (%)**			0.108
Folic Acid/Ferrous Fumarate	32 (91.4)	47 (97.9)	
Unknown	2 (5.7)	0	
No exposure	1 (2.9)	1 (2.1)	

Data are expressed as percentage. *P* value were calculated using χ^2 test for categorical values. PDA, Patent Ductus Arteriosus, DM T1, Diabetes mellitus type 1, DM T2, Diabetes mellitus type 2, H, Hypertension, RA, Rheumatoid arthritis, UTI, Urinary Tract Infection, VC, Vaginal candidiasis, NSAIDs, Nonsteroidal anti-inflammatory drugs.

A)

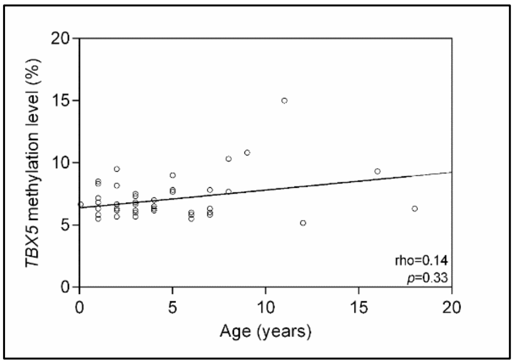

B)

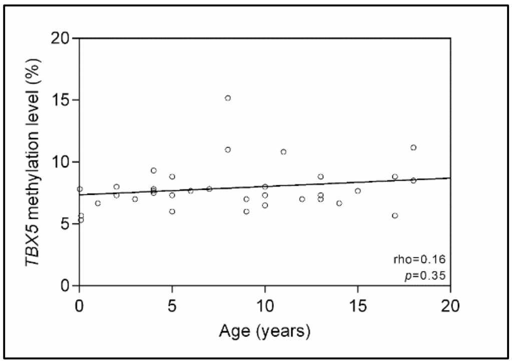

C)

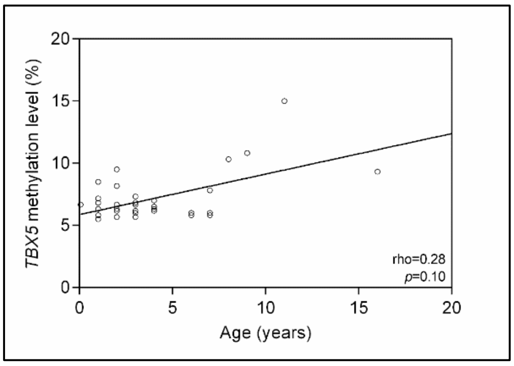

D)

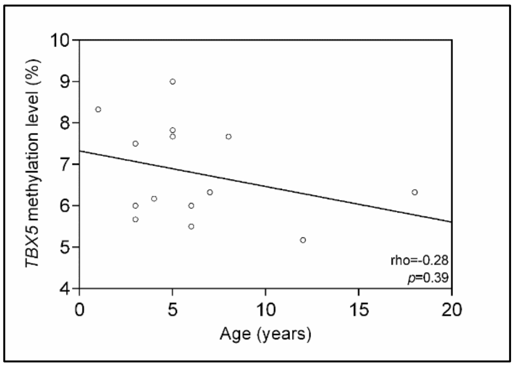

E)

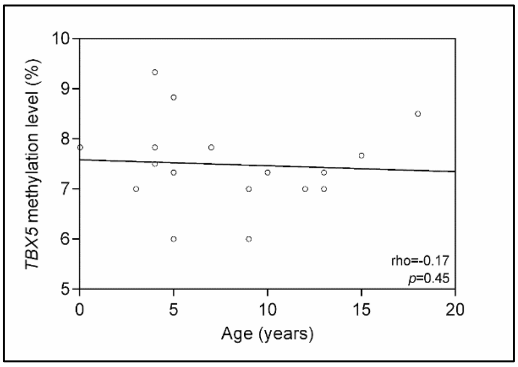

F)

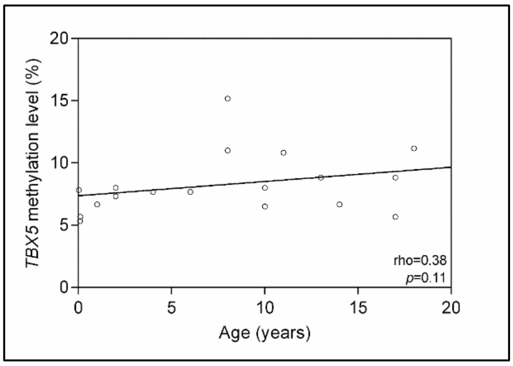

Supplementary Figure S1. Correlation of methylation levels (%) in the *TBX5* gene promoter and age expressed in years in the population study. **A)** Controls; **B)** Congenital Septal Defects patients; **C)** Women, controls; **D)** Men, controls; **E)** Women, Congenital Septal Defects patients; **F)** Men, Congenital Septal Defects patients. (Spearman).
